# Assessing the determinants of government spending efficiency in Africa

**DOI:** 10.1186/s43093-022-00142-8

**Published:** 2022-10-08

**Authors:** Abiodun Adegboye, Olawale Daniel Akinyele

**Affiliations:** Department of Economics, Obafemi Awolowo University, Ile-Ife, Nigeria

**Keywords:** Government spending, Efficiency, Human development, Natural resources, H53, H21, I30, Q28

## Abstract

Government spending is one of the vital ways for the provision of public goods and services with a view of improving citizens' well-being. African countries have been identified by international bodies as naturally endowed with resources that serve as major financiers for many African governments yet, most countries in Africa are ranked low in human development. Though the nature of many governments is hinged on the quality of life, however, the reverse is the case for many African countries. Low development indicators as against huge African governments spending indicates low efficiency in spending. Hence, this study assesses the efficiency of government spending in Africa and examines the drivers of government spending efficiency. Adopts SFA to assess government spending efficiency while TFE model was used to examine the relationship between government spending efficiency and its drivers. Owing to macrodata adopted in the study, it accounts for the second-generation panel unit root and uses panel corrected standard error to correct for cross-sectional dependence among 40 African countries between 2000 and 2020. The frontier result revalidates government spending as an input factor to achieve growing human development in Africa. The result shows that the level of government spending efficiency depends on the size of the economy and other factors. Natural resources could be used to address the burgeon government spending efficiency when effectively utilized. The result shows that colonial legacy has a long-lasting impact on government spending efficiency. These results suggest the need for efficiency of government spending owing burgeon drivers available among African economies. We recommend the need to improve the efficiency of government spending in order to situate framework for Africa development. Effective resources utilization and a strong institutional framework are potential drivers of spending efficiency in African economies. The paper provides an empirical study on the relationship between natural resources, colonial legacy, and government spending efficiency through true fixed effect among African countries.

## Introduction

**Fig. 1:**
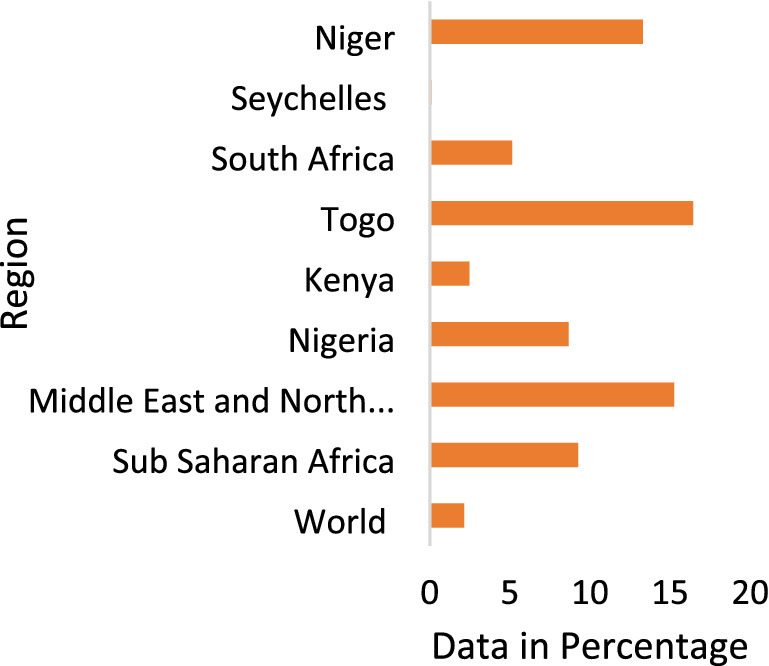
Total natural resources rent in percentage of GDP as at 2017

The performance of any government is measured by improvement in its citizens' well-being and the quality of life they live. Since the late 1930s, the government's involvement in economic activities has remained substantial and cannot be overemphasized [85]. Government spending has remained an important instrument for the provision of public goods and services including health, education, transport infrastructure, and security [13, 32, 54, 56, 59, 61]. Several analytical and empirical studies have focused on expenditure as the traditional avenue through which the government can achieve its aim [19, 22, 60]. Average government spending has remained high across African countries (see Table 1 and Figures), and this has been done to make public goods or services available (Fig. 1).

**Table 1:** Trend of Government Spending in Africa

YEAR	NAC	SAC	CEN	WAC	EAC
2000	13.62794	19.92787	11.55273	12.92683	14.49767
2001–2005	14.13163	18.80704	12.03282	12.11712	14.70728
2006–2010	13.93082	19.69506	10.71069	11.68656	12.944
2011–2015	16.72415	21.98794	12.73183	12.69992	14.82657
2016–2020	17.79961	24.95169	10.84884	12.31977	14.95413

*NAC* North Africa Countries, *SAC* Southern African Countries, *CEN* Central African Countries, *WAC* West African Countries, *EAC* East African Countries

**Fig. 2:**
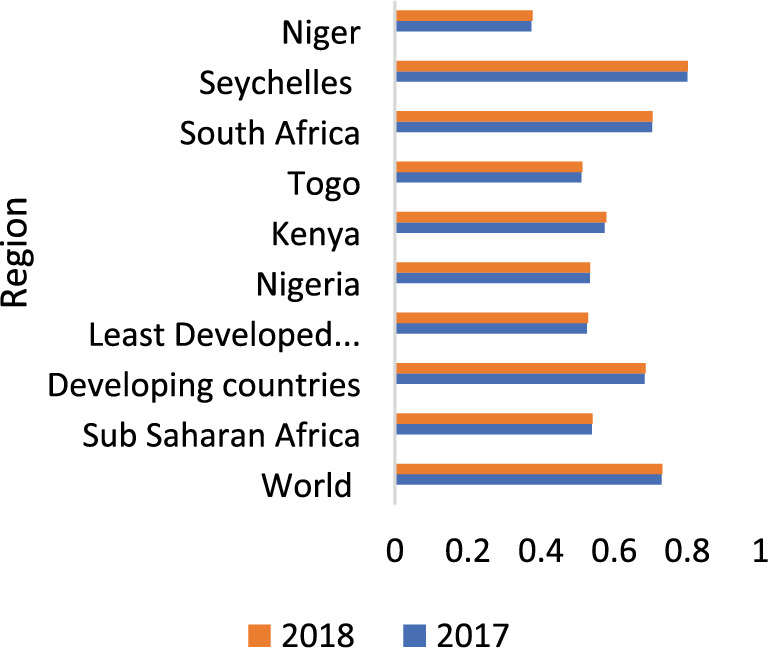
Human Development Index Trend

**Fig. 3:**
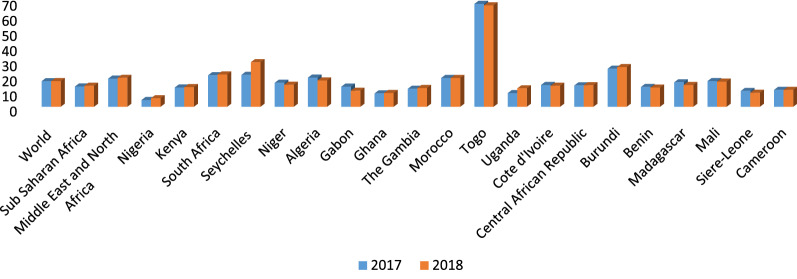
Government Expenditure as a Percentage of GDP

Government spending is one of the vital ways for the provision of public goods and services with a view of improving citizens' well-being. African countries have been identified by international bodies as naturally endowed with resources that serve as major financiers for many African governments yet, most countries in Africa are ranked low in human development [103]. Though, the nature of many governments is hinged on the quality of life, however, the reverse is the case for many African countries (see Figs. 2 and 3). Over the years, there has been an increase on average in the government spending of African countries owing to peculiar attributes in influencing economic productivity and well-being of the populace, yet her socio-economic outcomes seem to not match the amount or level of the spending. One critical question is to ask whether the efficiency level in the use of government spending to improve social welfare is increasing over time or not? In other words, the effectiveness of government spending in Africa may have been hampered by the quality of the spending. Hence, the paper examines the efficiency of African countries' government spending in the relationship between government spending and socio-economic outcomes as the framework (Single-output multiple-input approach), and the determinants of the level of efficiency in government spending, with particular attention to natural resources and colonial legacy across the sampled countries.

There is a need to improve socio-economic outcomes, especially among African countries where there exists the highest number of people living in poverty [110] and the high prevalence of food insecurity, (for example, increase from 54.3% in 2014 to 57.2% in 2015 for sub-Saharan Africa [6]. Among many factors, to improve socio-economic outcomes in Africa, it is paramount to increase the spending of government or secure higher efficiency in the use of the spending instrument or both. However, regardless of the choice made, an understanding of the efficiency of government spending is inevitable. More so, the tax base limits the extent to which the government can increase its expenditure pattern and there are other (macro) economic constraints to government effort towards increasing expenditure [57, 69].

In light of this, to have an effective and efficient provision of goods and services (social goods) in Africa, the government must improve the quality of spending, calling for higher efficiency. Herrera and Pang [50] noted that some countries with high expenditure patterns recorded a low level of efficiency in government spending. These countries include Angola, Niger, Sudan, Ethiopia, and Burkina Faso. Therefore, a small change in the factor(s) that accounts for a low level of government spending efficiency may have a significant impact on the socio-economic outcomes. The empirics on the level of efficiency of government expenditure in Africa show that most countries have low levels of efficiency in the utilization of public resources [2, 33, 43, 47, 50, 57, 69, 77].

Africa has an abundance of natural resources, experienced colonization for several decades, and has witnessed a varying level of institutional quality, which are some of the factors that may influence the efficiency level in government spending. Some authors have examined government efficiency and its underlying variations. Studies [2–5, 43, 78] focused on sectoral analysis. Likewise, income level produces cross-country efficiency variation [2, 3, 43, 99, 100], per capita income and government revenue caused variations inefficiency [48], gross domestic product (GDP) per capita, institutional structure and framework [70], urbanization and bureaucratic quality influence on the efficiency of spending [50, 57] whereas corruption, the incidence of human immunodeficiency virus or acquired immunodeficiency syndrome (HIV/AIDS) outbreak, higher-income inequalities, and the extent of external support financing are reported to have negative effects [50, 57]. However, the assessment of government spending efficiency in Africa or SSA may be incomplete, inaccurate, and unreliable if factors such as natural resources endowment, colonial legacy, and size of the economy are not included or accounted for. Moreover, studies at various times and spaces have identified the role of institutional quality towards government activities especially among countries of Africa, [10, 20, 21]. Hammond's [46] findings show that institutional variables have a direct and significant impact on government activities.

Many countries in Africa are natural resource-rich and operate in a democratic environment yet their level of development or socio-economic attainment remains low. Studies over the years have revealed that there is no agreement on the nexus between natural resources endowment and level of economic development [78–93]. As for Africa, a large number of resource-dependent countries are low in economic development. The countries that operate in a resources-rich democratic environment should seek to convert their assets both human and physical capital to support, enhance, and promote socio-economic outcomes. According to Venables [107], 12.2% of the world's oil production is attributed to Africa in 2010 and a lower percentage of 9.4% in 2014. Resource revenue has remained the larger part of budget revenue estimates in most of these countries and accounts for between 60 and 70% in some countries. Ironically, Africa's rich natural resources-dependent countries have low ratings in health and education outcomes (see Figs. 1 and 2). Natural resources endowment should be synonymous with development. Africa is richly endowed and seems to be most naturally blessed in the world, yet development outcomes are among the least, comparatively.

Similarly, the system of public sector operations and bureaucratic administrations has ever remained the reflection of what the colonial masters handed down. Colonial legacy has remained an important variable in the systems of African countries, from the operations of the systems of the public services, education, judiciary, financial institutions, and a host of others. Except for a few countries such as Rwanda, the legacy of colonization in official languages still guides the running of affairs in most African countries which had it. According to Agbor [11], colonial legacy may have indirect effects on socio-economic outcomes via some channels, though the direct effect would be treated with caution. Therefore, the measure of colonial origin would be factored as a possible determinant of the level of efficiency or as affecting the extent of economic development in Africa. Hence, the study probe whether colonization hinders government spending efficiency in African countries or not? The kernel of this study is therefore to examine the main factors influencing the level of efficiency in public spending in Africa. Some related studies [2, 33, 50, 57, 69, 77] that focused on determinants of the efficiency of government spending in Africa are mainly on specific sectors but neglected the roles of natural resources endowment as well as the influence of colonization within a parametric frontier analysis.

Following the introduction, the study discusses the related existing studies on the efficiency of government spending and its underlying variations in Section two. In Section Three, we present the methodological approaches adopted in the study. In Section Four, results and discussion of the study are presented and Section five discusses the conclusion and policy implications.

## Review of related literature

### Theoretical foundations

The paradigm of government spending efficiency can be anchored on the Keynesian proponents in African economies. Government spending has been argued to have a positive relationship with well-being, following insights from Wagner [109], Peacock and Wiseman [80], Musgrave [71], and the argument from various empirical standpoints [54, 85, 87]. Specifically, the Wagner law of state activity emphasizes an endogenous relationship, because as an economy is growing likewise government spending is increasing and the increase in government spending on social progress will make the economy increase in; education, public health, old age pension or retirement insurance, food subsidy, environmental protection programmes, natural disaster aid, and other welfare functions in turn. However, a cursory look at the socio-economic outcomes in Africa shows that reverse is the case between growing government spending and socio-economic outcomes. Countries that are ranked first in development episode among African countries were among the least in the world [103]. Low development indicators as against huge African governments spending indicates low efficiency in spending. In order to answer how efficient in the use of government spending to improve social welfare, the study is classified into two phases. In the first phase where frontier approach was adopted to establish the government spending efficiency level, government becomes producer of social welfare through the output–input approach. This explain the efficiency of government spending through single output (human development index) and multiple inputs (government spending, labour productivity, and gross fixed capital formation). The second phase assesses the determinants of government spending efficiency by establishing static relationship between government spending efficiency and its core drivers.

### Empirical evidence

The need for greater efficiency has remained a key interest of policymakers and researchers in the discussion of government spending, especially among African countries where development issues of rising poverty, wide inequality, and high unemployment are more pronounced, despite a consistent rise in government expenditure. One of the ways of addressing the problems, as used in the literature, is via fiscal spending instruments.

Government spending has experienced an increase over time in tackling and addressing such development issues [13, 19, 22, 32, 35, 54, 56, 64–61, 85]. Despite growing spending, there is still a persistent high problem of poverty, inequality, and unemployment. Meanwhile, studies show that government spending has been very much below the required amount due to some reasons including narrow tax bases as well as structural macroeconomic constraints, [57, 69]. Thus, an examination of government spending efficiency and its determinants becomes inevitable and may have been a key factor in explaining the status of socio-economic outcomes observed in Africa.

Several studies across developing regions have provided evidence that abundant natural resources do not equate to economic development [14, 36, 49, 89–107]. Africa is blessed with an abundance of both natural and non-natural resources, yet its development indices are among the least in the world. Hamdi and Sbia [45] explained that resources endowment remains the principal source for growth and the main channel with which to finance government spending. Dizaji [30] examined the dynamic relationship between natural resources and government spending with evidence showing that the government's oil export can potentially affect government total expenditures. Ahmad and Masan [7] investigated the short-run and long-run relationships between the real GDP, the real government expenditure, and the real oil resources for the period between 1971 and 2013. They found a positive long-run relationship between the variables in the study. Mahmoodi [66] explored oil price reduction impacts on the Iranian economy and showed that oil export and the mineral commodity export earnings decreased, but other production sectors' exports increased. Musau and Veka [72] studied the nexus between crude oil trade and current account deficits by building on and extending Huntington [112]. Hence, the contributions of the gain realized from resource rent to government spending efficiency becomes imperative, since African countries are characterized by an abundance of natural resources.

No doubt, resources endowment influences the direction of a country’s economic activities, particularly within the region that shares common trade, language, and geographical location. Glyfason’s [39] study revealed that natural resources impede economic growth through the crowding out of foreign and social capital. Natural resources endowment remains part of the major sources that can be used to finance expenditure. Natural resources are generally government-controlled, and hence, its contribution to the provision of social goods cannot be overemphasized. The question as to whether natural resources are a blessing on economic growth and development is an imperative issue and requires clarification. Kim and Lane [59], using a sample of developing countries, reported that economies endowed with abundant natural resources tend to develop more slowly than the ones with scarce resources. They concluded that natural resources are, on average, a curse for developing countries. Over the years, studies have also examined the role and economic advantage of natural resources, across countries. Natural resources abundance may crowd out manufacturing activities [67, 92, 101], lead to underinvestment in human capital [38, 44], cause rent-seeking behaviour [17, 102], increase incidences of social conflict and civil war [29, 51], and undermine institutional quality [1, 15]. Ever since the work by Sachs and Warner [90], the finding that natural resources appear to be more of a curse than a blessing has led to the extensive literature. Owing to the nature of endowed natural resources as government-controlled, it becomes plausible to examine the efficiency of government spending in light of natural resources endowment.

Furthermore, owing to a government measure of performance, studies have provided a testable proposition on the nexus between government spending and socio-economic outcomes. Peacock and Wiseman's theory of public expenditure argued that public expenditure does not follow an organic state but instead a step-like fashion. Explicitly, the government is interested mainly in ensuring the well-being of the populace and when the unexpected, such as war, disease outbreak, pandemic, and other shocks (positive or negative) occurs, the expenditure profile has to increase as a means of intervention to curtail the consequences of shock. Hence, in an attempt to maintain or improve social welfare, there is a need for government to increase spending which the existing government resources might not be adequate to achieve, as such resource-rich countries could achieve the objective of well-being during this period non-resource-rich countries. The implication of this theory on the need for efficiency is that given social/natural disturbances (positive or negative shocks) and the need to provide public goods, efficient use of such resources may help to facilitate human development and economic growth.

Also, Musgrave [73] asserted that the demand for public service tends to be very low when per capita income is low. The assertion is due to him, such income is dedicated to fulfilling primary needs, and when per capita income begins to rise above low-income levels, demand for the public sector provision of utilities such as health, education, and transportation begins to rise, forcing government spending on them to increase. Per capita income has a high correlation with public sector social service demand with supply. So, as Africa has experienced sustained growth in the last 30 years [except during corona virus disease (COVID-19) lockdown], the social demand for public goods has risen and the size of the public sector has been forced to increase its activities. One key implication is that pressure remains on the government to provide public goods, which the current state has remained behind the required amount, leading to the infrastructural deficit, high out of school children, poor health, etc., in Africa. The state of socio-economic outcomes in Africa is by far below expectations as a result of a deficit in these public goods, among others.

Unarguably, high quality of life may be achieved with increased government spending [54, 71, 80, 85, 87, 109]. Meanwhile, poverty continues to be prevalent, with large inter-country and intra-country disparities, particularly in West, East, Southern, and Central Africa as a group. Likewise, manufacturing and value addition in most countries in Africa remain weak due (in part) to constrained infrastructure and the prevalence of racially biased cultural norms that strengthen their behaviour [6, 103].

The core objective of the government is to promote well-being through allocation, distribution, stabilization, and economic growth, and as such government must spend. The quality of government spending in Africa has become more important due to the higher demand for public goods or services and uncertain external development assistance. Having experienced a growing expenditure pattern in Africa over the years with low government performance, the efficiency of government spending, therefore, becomes essential.

Studies have examined the determinants of government spending efficiency across the world. The range of studies includes the differences in the efficiency of education spending [3], public sector efficiency [3], determinants of expenditure efficiency [48], the efficiency of public expenditure in improving health, education and governance performance [86], assessment of the effectiveness of health care delivery [111], efficiency and variation in cultural and economic characteristic [40], Also, there was a re-estimation of efficiency with parametric and nonparametric methods [52]. Literature shows that bureaucratic quality and urbanization have a positive influence on the efficiency of spending whereas corruption, the incidence of HIV/AIDS outbreak, higher-income inequalities, and the extent of external support financing have a negative influence on public spending efficiency. Studies also see civil unrest as a crucial factor that determines the efficiency of public spending. The importance of natural resources rents on economic development cannot be overemphasized. The contribution of natural resources rent to economic development, have evolved in the literature overtimes. Natural resources abundance and financial development [97]. Mining revenues, government consumption, exchange rate, and economic growth [62]. Total natural resources, economic growth, financial development, capital stock, and trade openness, (Satti et al. 2014). GDP, natural resource rent, and trade openness, [45]. What is the role of natural resources endowment on the level of government spending efficiency?

The curse of natural resources refers to the paradox that resource-rich countries tend to grow slower than resource-poor countries, despite their large natural resources endowments [93]. Auty [16] argued that the slow economic growth that many Latin American, sub-Saharan African, and Arab countries have experienced during the 1970s is mainly because, instead of creating wealth and developing human capital, the associated governments just sought for gaining more political and popular support by distributing rents collected from natural resources exports.

Some studies have argued that natural resources abundance is a curse [18, 88] while some report it as a blessing [89, 108]. Havranek et al. [49] also showed that 40% of empirical studies published within the two last decades agreed that natural resources are a curse while 20% opined that it is a blessing and the last 40% of studies report no significant effect. There is no consensus on whether natural (resource) rent is a blessing or a curse. According to the World Bank [110], the report noted that natural resources as a curse are not universal since many countries both developed and developing such as Canada, Botswana, and Australia have succeeded in transforming their raw natural resources into sustainable development. Hence, this study will validate the natural resources-government spending efficiency nexus among African countries.

In terms of approaches employed to investigate the nexus in recent times, both parametric and nonparametric strategies have been dominant in the literature. However, a parametric approach such as stochastic frontier analysis (SFA) separates deviations from production function between random error and inefficiency [25]. The SFA was independently proposed by Aigner, Lovell, and Schmidt [9] as well as Meeusen and Van den Broek [68] to estimate technical efficiency in applied economic research. As for the nonparametric approach such as free disposal hull (FDH) and data envelopment analysis (DEA), what is involved is a mathematical programming technique and it allocates all the deviations from the production frontier to technical inefficiencies [65]. There is no specific functional relationship in the production frontier between inputs and output or in the efficiencies term, unlike the SFA.

The specific functional relationship between inputs and output in the SFA methodology to efficiency can be Cobb–Douglas, translog, or any other which may have been developed along the line of specific distributions and estimation methods. For instance, Battese and Coelli, [25] proposed truncated normal distribution and maximum likelihood for the estimation of inefficiency term in a panel analysis. Greene [41] provided an extension through the measure of efficiency as time-invariant. He incorporated a term used to capture the inefficiency for the random effects and fixed effects estimators containing time-invariant cross-unit heterogeneity.

## Methodology

Aigner et al. [9] and Meeusen and van den Broeck [68] presented the studies that introduced SFA in econometrics. Pitt and Lee [84], Jondrow et al. [58], Schmidt and Sickles [95], Battese and Coelli, [22–26], Kumbhakar [63], Greene [41, 42], and others have extended the original formulation to the models to suit various empirical analyses. The SFA modelling strategy on the one hand permits inference making on the determinants of the frontier of the functional variable. On the other hand, it explains the drivers of inefficiency therein. The possible sources of inefficiencies in government spending may be exogenously determined by factors that may not necessarily be within the control of the economic agents (such as the government). Therefore, every SFA model specification entails the inclusion of environmental/exogenous variables. The stochastic frontier model of government spending adopts single-output multiple-input approach that employs panel data is given as:

1 $$ Y_{it} = f\left( {X_{it} ;\beta } \right) $$

where *Y* is the maximum output obtainable (human development index), *X* is the vector of non-stochastic inputs (government spending, labour productivity, and gross fixed capital formation), and *β* is an unknown parameter. The SFA allows producer (government) to incorporate the possibility of measurement error into inefficiency term and noise effect as follows;

2 $$ \begin{aligned} Y_{it} = & X_{it} ;\beta + \varepsilon_{it} \;{\text{for}}\;i = 1, \ldots , N\;{\text{and}}\;t = 1, \ldots , T \\ Y_{it} = & X_{it} ;\beta + V_{it} - U_{it} \\ \end{aligned} $$

where $$\varepsilon_{it} = V_{it} - U_{it}$$.

Here, $V_{it} \sim i.i.d.$ $N\left( {0,\sigma_{v}^{2} } \right)$ and $U_{it} \sim i.i.d.$ $N\left( {\mu ,\;\sigma_{\mu }^{2} } \right)$ are identically and independently distributed of each other and other explanatory variables. *N* and *T* represent the number of countries in the sample and time period end, respectively. Following, Greene [41] as well as Battese and Coelli [25] models which assume truncated normal distribution, this study estimated the technical efficiency of government spending in selected forty (40) African countries through the Cobb–Douglas stochastic frontier function. The linear form of the equation, which is made up of the deterministic component and the composite error, is derived for estimation. However, the composite error is decomposed into noise effect, $V_{it}$ and inefficiency function, $U_{it}$. The technical efficiency (TE) of production for the *i*th country at time t is defined as:

3 $$ \begin{gathered} TE_{it} = \frac{{\exp (X_{it} \beta + V_{it} - U_{it} )}}{{\exp (X_{it} \beta + V_{it} )}} \hfill \\ TE_{it} = \exp (U_{it} ) \hfill \\ \end{gathered} $$

Note that the expected value of the exponential (− *U*_*it*_) represents technical efficiency. Given the truncated normal assumption in the stochastic frontier analysis, the technical inefficiency model is specified; thus,

4 $$ U_{it} = \delta_{i} Z_{it} + W_{it} $$

*Z*_*it*_ represents the environmental/ exogenous variables (government spending, size of an economy, resource rent, colonial legacy, and institutional variables) that drive the efficiency of the frontier. The choice of explanatory variables is guided by economic theory, particularly the Keynesian proponents. *W*_*it*_ is the error term of the efficiency model and is the estimated parameter.

The study utilizes a panel data of 40 African countries (Algeria, Egypt, Morocco, Sudan, Tunisia, Botswana, Namibia, South Africa, Swaziland, Angola, Cameroun, Central Africa Republic, Chad, Congo Brazzaville, Congo DR, Gabon, Benin, Burkina Faso, Gambia, Ghana, Guinea, Guinea Bissau, Mali, Niger, Nigeria, Senegal, Sierra Leone, Togo, Mauritania, Burundi, Comoros, Kenya, Madagascar, Mauritius, Mozambique, Rwanda, Tanzania, Uganda, Zambia, and Zimbabwe) owing to the international monetary fund (IMF) [55] and Venable [107] and covering the period between 2000 and 2020 following the era of strong growth in output and increased economic activities among most African countries. Data were sourced from 2021 Editions of the World Bank's Development and Governance Indicators to examine the relationship between government spending efficiency and its possible drivers such as natural resources and others using five different models. Following Greene [41], the true fixed model was considered as baseline because allows different distributional assumptions, providing the modelling of both inefficiency location and scale parameters. In addition, capture the inefficiency for the fixed effects estimators containing time-invariant cross-unit heterogeneity. The variables and measures in the model are presented in Table 2.

**Table 2:** Data and Sources. *Source* Authors Compilation (2022)

Variables	Symbols	Measurement	Source
Human development index	HDI	This is a statistic composite of human development measured by life expectancy, literacy rate and per capita income which represent the single output of the frontier	WDI
Government spending	GOV	This is the ratio of government expenditure to GDP	WDI
Size of an economy	GDP	This is measured by GDP (current US$)	WDI
Resource rent	RENT	This is measured by total natural resources rents as a % of GDP	WDI
Institutional variables	COR	This is measured by control of corruption	WGI
	EFFE	This is measured by government effectiveness	WGI
Colonial legacy	COL	This is measured by the inception of country independent through the study period	Authors' calculation
Dummy variable	DUM_RE	This is measured as ''1'' for a resource-rich country and "0" for non-resource-rich following IMF classification	Authors' construct
Government spending efficiency	EFF	This is measured by the Stochastic Frontier with single output-multiple input	Authors' construct
Gross fixed capital formation	CAP	This is measured as gross fixed capital formation (% of GDP) which represent part of the multiple input of the frontier	WDI
Labour productivity	LAB	Labour force participation rate (% of total population ages 15 +) which represent part of the multiple input of the frontier	WDI

## Results and discussion

This section provides a summary of the result on government spending efficiency in African countries. This section is divided into three subsections. In subsection one is the descriptive, correlation and pretest of the model while subsection two presents the frontier result of government spending efficiency and finally, subsection three presents the drivers of government spending efficiency across the sample frame.

### Descriptive, correlation and pretest

The empirical results within the government spending efficiency start with the descriptive statistics to have a clearer view of the series and the cross sections used in the study. Among the selected countries in Africa, Table 3 reveals that most human development countries stood at 0.804 representing very high human development following the UNDP Report [103] while on average human development for Africa stood at 0.502 representing low human development. By implication, most African countries' human development falls within low human development suggesting the need for African countries to improve the well-being of the region. Meanwhile, government spending in the region on average stood at 14% though some countries spend as high as 35% government spending on economic activities in the region. Impliedly, achieving well-being is seen as the paramount objective for the African government with huge government resources yet human development is within the lower echelon among countries of the world. Furthermore, at most 59% were revenue mobilized through rent resources among African countries while on average African countries generates 11% of their revenue base from rent resources. This could be the reason for international bodies (IMF, UNDP, World Bank) identified most of the countries in the region as resource-rich countries [55, 107]. Likewise, Table 4 reveals a positive degree of association between human development and government spending. This is not unexpected as suggested by theories and empirical standpoint [12]. However, there is an indirect association between human development and resources rent among African countries in the study. Also, the degree of association between variables and the coefficient at the diagonal showed the degree of association between a variable and itself. Following Bolarinwa et al. [27], the degree of association between variables is within the range of the acceptance region and the result are not unexpected since it conforms to theoretical foundation of Keynesian proponents of our a priori expectation. Owing to macropanel data adopted in the study, it becomes celestial to carry out pretest to avoid the misleading result.

**Table 3:** Descriptive statistics

Variable	Obs	Mean	Std.Dev	Min	Max
HDI	840	0.502	0.116	0.252	0.804
GOV	840	14.028	5.125	.952	35.351
GDP	840	4.03e + 10	8.24e + 10	3.51e + 08	5.47e + 11
LAB	840	64.863	13.025	40.3	89.05
CAP	840	21.778	8.745	1.097	81.021
RENT	840	11.005	10.499	.001	58.65
COR	840	-0.648	0.563	− 1.57	1.22
EFFE	840	-0.696	0.602	− 1.88	1.06
COL	840	46.575	12.337	6	98
DUM_RE	840	0.575	0.495	0	1
EFF	840	0.968	0.027	0.811	0.993

*HDI* human development index, *GOV* government spending, *GDP* size of an economy, *LAB* labour productivity, *CAP* gross fixed capital formation, *RENT* resources rent, *COR* control of corruption, *EFFE* government effectiveness, *COL* colonial legacy, *DUM_RE* dummy variable, *EFF* efficiency

**Table 4:** Matrix of correlations

Variables	(1)	(2)	(3)	(4)	(5)	(6)	(7)	(8)	(9)	(10)	(11)
(1) HDI	1.000										
(2) GOV	0.276	1.000									
(3) GDP	0.385	− 0.056	1.000								
(4) LAB	− 0.470	− 0.047	− 0.285	1.000							
(5) CAP	0.264	0.145	0.004	− 0.144	1.000						
(6) RENT	− 0.098	− 0.144	− 0.004	0.081	0.199	1.000					
(7) COR	0.453	0.386	0.047	− 0.193	0.087	− 0.479	1.000				
(8) EFFE	0.571	0.349	0.175	− 0.182	0.136	− 0.438	0.864	1.000			
(9) COL	0.166	− 0.214	0.096	− 0.160	0.191	0.041	− 0.170	− 0.153	1.000		
(10) DUM_RE	− 0.212	− 0.199	− 0.057	0.222	0.363	0.431	− 0.331	− 0.342	0.159	1.000	
(11) EFF	0.121	− 0.237	0.196	− 0.045	0.022	0.028	− 0.169	− 0.190	0.541	0.104	1.000

*HDI* human development index, *GOV* government spending, *GDP* size of an economy, *LAB* labour productivity, *CAP* gross fixed capital formation, *RENT* resources rent, *COR* control of corruption, *EFFE* government effectiveness, *COL* colonial legacy, *DUM_RE* dummy variable, *EFF* efficiency

To analyse the presence of cross-sectional dependence and the order of integration of our variables, the Pesaran cross-sectional dependence (CD) test [82], and the cross-sectionally augmented IPS (CIPS) test [83] were performed. Tables 5 and 6 give the results of both tests. Given the results of the Pesaran CD test [82] in Table 5, there is a presence of cross-sectional dependence in all the variables among selected African countries. By implication, African countries are correlated in terms of series adopted for the study. The reason could be common continental attributes (*particularly decolonized public sector arrangement*) that most of the countries shared and as such if we ignore it, it can produce inconsistent and incorrect conclusions in the econometric approach [31]. Within the foregoing, since there is the presence of cross-sectional dependence in the series, second-generation unit root test, the CIPS test by Pesaran [83] becomes bodacious because first-generation unit root tests are not trustworthy when this phenomenon is present. Table 6 reveals that some of the variables are on the borderline between the orders of integration *I*(0)/*I*(1), but that in the first differences, all variables are stationary with and without trend.

**Table 5:** Cross-sectional dependence

Variable	CD-test	*p*-value	Corr	Abs(corr)
GOV	6.880	0.000	0.054	0.422
GDP	110.530	0.000	0.864	0.864
RENT	33.620	0.000	0.263	0.377
COR	0.870	0.384	0.007	0.332
EFFE	5.810	0.000	0.045	0.391
EFF	104.040	0.000	0.813	0.813

Under the null hypothesis of cross-section

Independence CD ~ *N*(0,1)

*GOV* government spending, *GDP* size of an economy, *RENT* resources rent, *COR* control of corruption, *EFFE* government effectiveness, *EFF* efficiency

**Table 6:** Second generation unit root

(A) Maddala and Wu (1999) Panel unit root test (MW)			
Variable	Specification	Without	Trend
	Lags	chi_sq	*p*-value
EFF	0	360.302	0.000
EFF	1	205.637	0.000
GOV	0	86.715	0.285
GOV	1	78.748	0.519
GDP	0	34.452	1.000
GDP	1	51.178	0.995
RENT	0	94.817	0.123
RENT	1	105.556	0.029
COR	0	107.788	0.021
COR	1	139.188	0.000
EFFE	0	134.466	0.000
EFFE	1	208.649	0.000
COL	0	0.000	1.000
COL	1	0.000	1.000
DUM_RE	0	0.000	1.000
DUM_RE	1	0.000	1.000

Variable	Specification	With	Trend
	Lags	chi_sq	*p*-value
EFF	0	92.931	0.153
EFF	1	71.653	0.736
GOV	0	89.110	0.228
GOV	1	102.225	0.048
GDP	0	73.624	0.679
GDP	1	55.412	0.984
RENT	0	61.635	0.937
RENT	1	93.721	0.140
COR	0	68.298	0.822
COR	1	107.732	0.021
EFFE	0	110.489	0.014
EFFE	1	172.627	0.000
COL	0	0.000	1.000
COL	1	0.000	1.000
DUM_RE	0	0.000	1.000
DUM_RE	1	0.000	1.000

(B) Peseran (2007) Panel unit root test (CIPS)				
Variable	Specification	Without		Trend
	Lags	Zt-bar	*p*-value	*t*-bar
EFF	0	− 4.427	0.000	
EFF	1	− 3.855	0.000	
GOV	0	0.854	0.803	
GOV	1	1.423	0.923	
GDP	0	1.204	0.886	
GDP	1	0.916	0.820	
RENT	0	− 2.732	0.003	
RENT	1	− 2.868	0.002	
COR	0	0.399	0.655	
COR	1	− 0.321	0.374	
EFFE	0	− 2.724	0.003	
EFFE	1	− 2.438	0.007	
COL	0	28.493	1.000	
COL	1	28.493	1.000	
DUM_RE	0	28.493	1.000	
DUM_RE	1	28.493	1.000	

Variable	Specification	With	Trend	*t*-bar
	Lags	Zt-bar	*p*-value	
EFF	0	− 3.154	0.001	
EFF	1	− 2.693	0.004	
GOV	0	− 0.406	0.343	
GOV	1	− 0.291	0.385	
GDP	0	− 3.421	0.000	
GDP	1	− 4.396	0.000	
RENT	0	1.011	0.844	
RENT	1	− 0.070	0.472	
COR	0	2.451	0.993	
COR	1	2.050	0.980	
EFFE	0	− 1.729	0.042	
EFFE	1	− 0.863	0.194	
COL	0	27.270	1.000	
COL	1	27.270	1.000	
DUM_RE	0	27.270	1.000	
DUM_RE	1	27.270	1.000	

Null for MW and CIPS tests: series is I(1)

MW test assumes cross-sectional independence

CIPS test assumes cross-sectional dependence is in form of a single unobserved common factor. -multipurt- uses Scott Merryman's -xtfisher- and Piotr Lewandowski's -pescadf-

*GOV* government spending, *GDP* size of an economy, *RENT* resources rent, *COR* control of corruption, *EFFE* government effectiveness, *COL* colonial legacy, *DUM_RE* dummy variable, *EFF* efficiency

### Frontier analysis

In this subsection, government spending efficiency was generated through a parametric approach of the stochastic frontier method owing to the underlying assumption of the functional relationship as explained in the methodology across the sample frame. In a conventional production approach, the production process involves different combinations of inputs that gives output and as such, the performance of any decision-making unit can be quantified within this framework. In this study, government spending efficiency was generated using human development as the output variable of the frontier while input factors used to generate government spending efficiency were government spending, gross fixed capital formation and labour productivity following the Cobb–Douglas production function. Meanwhile, to enhance our understanding of the need for government spending efficiency among African countries, the study breaks the sample size into resource-rich and non-resource-rich countries as a robustness check and we established government spending efficiency among resource-rich and non-resource-rich African countries following IMF [55] and Venables [107] classification of resources-rich and non-resources countries. Table 7 presents the baseline and robust models across the sample size. Government spending, gross fixed capital formation and labour productivity are highly significant in explaining government spending efficiency across the sample size. The results show that government spending and capital formation are positively and significantly influence human development among African countries. Meanwhile, there is a negative relationship between labour productivity and human development. Thus, these signs are not unexpected since it is in tandem with a priori expectation and theoretical foundation of Keynesian proponents [37, 79, 81, 96, 98]. The frontier result shows that there is wide variation in the use of input factors among countries in Africa to achieve human development. However, the estimated gamma in Table 7, following Bolarinwa et al. [27] study shows that the total variation in human development is explained by technical inefficiency. Hence, this serves as a good proxy of government efficiency. Meanwhile, other diagnostic checks are in the right magnitude.

**Table 7:** Frontier model for government efficiency. *Source* Authors Computation (2022)

Dep: HDI	Coefficient	Dep: HDI	Coefficient	Dep: HDI	Coefficient
Frontier		Frontier		Frontier	
African Countries		Resource-rich African Countries		Non-resource-rich African Countries	
GOV	0.003(0.001)***	GOV	0.004(0.0008)***	GOV	0.006(0.0009)***
LAB	− 0.009(0.001)***	LAB	− 0.014(0.0008)***	LAB	− 0.0069(0.001)***
CAP	0.001(0.000)***	CAP	0.002(0.0003)***	CAP	0.002(0.0006)***
Diagnostics		Diagnostics		Diagnostics	
Mu	0.899(0.969)	Mu	3.882(1.928)**	Mu	0.9559(0.968)
Log likelihood	1589.0268	Log likelihood	995.2863	Log likelihood	650.6517
Prob. Value	0.0000	Prob. Value	0.0000	Prob. Value	0.0000
Sigma_*U*^2^	0.1584	Sigma_*U*^2^	0.1385	Sigma_*U*^2^	0.1309
Sigma_*V*^2^	0.000096	Sigma_*V*^2^	0.0004	Sigma_*V*^2^	0.000625
Gamma	0.9277	Gamma	0.9475	Gamma	0.9354

*HDI* human development index, *GOV* government spending, *LAB* labour productivity, *CAP* gross fixed capital formation

Significant values at 1%, 5% and 10% for ***, ** and * respectively

The result shows that governments of the African countries have not been producing on the frontier level, as earlier found in previous studies [50]. However, the deviation in error term is due to technical inefficiency in the resources utilization effort of the government. Having established that governments of African countries are inefficient in the use of government spending as reported in some studies [50, 75], existing studies have made provision for the approach to the stochastic frontier analysis of inefficiency estimation [23, 53, 58]. Based on efficiency measures, we provided efficiency scores following [23, 58]. Table 3 reveals government spending efficiency for African countries has a high level of consistency given that their mean and median values are in between their maximum and minimum values across the descriptive statistics. Also, the standard deviation values showed that the series employed by the study are not different from their mean values

### Drivers of government spending efficiency

Having established the basic behaviour and stochastic frontier of government spending efficiency, we ran five different models to establish the drivers of government spending efficiency among African countries. Following Greene [41], the true fixed effect (TFE) model was used as a baseline model while true fixed effect model (TRE), true random effect model (TRE), panel corrected the standard error, and generalized ordinary least square (GLS) model were used as robustness checks to establish relationship among government spending efficiency and its determinants. Table 8 establishes a relationship between government spending efficiency and its drivers for selected African countries used in the study while Table 9 presents government spending efficiency and its drivers for resource-rich and non-resources African countries.

**Table 8:** Drivers of government spending efficiency in African countries

DEP VAR: EFF	DEP VAR: government spending efficiency					
	TFE	TFE_NRC	FEM	REM_NRC	PCSE	PCSE_NRC
GOV	− 0.019 (0.040)	− 0.0592 (0.041)	− 0.002 (0.000)***	− 0.0003 (0.0002)	− 0.001 (0.0002)***	− 0.001 (0.0002)***
GDP	0.000 (0.000)***	0.000 (0.000)**	− 0.000 (0.000)	0.000 (0.000)***	0.000 (0.000)***	0.0000 (0.000)***
COR	1.119 (0.717)	0.028 (0.893)	0.0051 (0.0038)	0.0031 (0.0039)	0.0073 (0.0024)***	0.0012 (0.002)
EFFEC	− 1.837 (0.677)***	− 1.633 (0.623)***	0.0074 (0.004)**	− 0.0152 (0.004)***	− 0.0074 (0.0021)***	− 0.0086 (0.002)***
RENT	− 0.021 (0.022)		0.0001 (0.0001)		0.00008 (0.00008)	
COL	0.112 (0.015)***		0.0029 (0.000)***		0.0014 (0.0002)***	
DUM_RE	− 0.014 (0.516)				− 0.0036 (0.0022)	
Cons	− 0.899 (0.969)	3.645 (0.966)***	0.8568 (0.005)***	0.962 (0.004)***	0.9164 (0.011)***	0.9742 (0.006)***
No of Group	40	40	40	40	40	40
Obs. Per Group	21	21	21	21	21	21
Wald Chi	256.80***	207.18***		19.66***	91.70***	50.48***
No of Obs	840	840	840	840	840	840
F-statistic			166.08***			
R-Square			0.557	0.166	0.991	0.9911
Haussmann			252.58***	3.96		
CS Dependence			26.757***	77.132***		

*TFE* total fixed effect, *FEM* fixed effect model, *REM* random effect model and *PCSE* panel corrected standard error, while *NRC* represent models without resource rent and colonial orientation. *GOV* government spending, *GDP* size of an economy, *RENT* resources rent, *COR* control of corruption, *EFFE* government effectiveness, *COL* colonial legacy, *DUM_RE* dummy variable, *EFF* efficiency

*** and ** represent 1% and 5% levels of significance with standard error in parenthesis

**Table 9:** Drivers of government spending efficiency in resource-rich and non-resource-rich region

DEP VAR: EFF	RESOURCE-RICH			NON-RESOURCE-RICH		
	TFE	REM	PCSE	TFE	FEM	GLS
GOV	− 0.005 (0.065)	− 0.002 (0.0003)***	− 0.002 (0.0003)***	− 0.027 (0.039)	− 0.004 (0.0003)***	− 0.003 (0.0002)***
GDP	0.0000 (0.0000)	0.0000 (0.000)	0.0000 (0.000)*	0.0000 (0.0000)**	− 0.000 (0.000)***	0.000 (0.000)***
COR	− 1.820 (0.606)***	− 0.011 (0.005)**	− 0.009 (0.004)**	0.777 (0.342)**	0.023 (0.004)***	0.004 (0.002)**
EFFEC	0.150 (0.028)***	0.003 (0.0002)***	0.002 (0.0003)***	0.105 (0.019)***	0.004 (0.000)***	0.001 (0.000)***
RENT	− 0.022 (0.029)	− 0.0002 (0.0002)	0.0002 (0.0001)	0.095 (0.046)**	0.0003 (0.0003)	0.0003 (0.0002)
COL			0.8734 (0.016)***			
Cons	− 3.882 (1.928)***	0.865 (0.011)***		− 0.956 (0.968)	0.852 (0.008)***	0.936 (0.008)***
No of Group	23	23	23	17	17	17
Obs. Per Group	21	21	21	21	21	21
Wald Chi	317.59***		123,828.60***	126.64***		140.00***
No of Obs	483	483	483	357	357	357
F-statistic					193.95***	
R-Square		0.4004	0.9803		0.7432	
Haussmann		5.76			312.26***	
CS Dependence		13.906***			7.218***	

*TFE* total fixed effect, *FEM* fixed effect model, *REM* random effect model, *PCSE* panel corrected standard error, *GLS* generalized least square, *GOV* government spending, *GDP* size of an economy, *RENT* resources rent, *COR* control of corruption, *EFFE* government effectiveness, *COL* colonial legacy, and *EFF* efficiency

***, **, and * represent 1%, 5%, and 10% levels of significance with standard error in parenthesis

In Table 8, TFE model was implemented as the baseline for drivers of government spending efficiency. To control for unobserved characteristics and cross-sectional dependence among African countries in the study, TFE, TRE, and PSCE were implemented. The result from Haussmann test shows that FEM is the appropriate model for unobserved attributes among African countries though there is cross-sectional dependence. For the sensitivity of the result, the model without rent resource and colonial legacy implement TRE following the Haussmann result. Also, to control for cross-sectional dependence, the PSCE model was implemented because the number of countries in the study is greater than the period. The Wald Chi and F-statistic result show the models are good for predicting the relationship between government spending efficiency and its drivers.

The results show that government spending has a negative but insignificant influence on the efficiency level. By implication, as government spend more, the efficiency level is reduced indicating low human development is achieved with high government spending instrument among African countries. Meanwhile, the robust models reveal a negative and significant relationship between government spending and efficiency levels among African countries. The negative and significant relationship between government spending and efficiency level in Africa is in tandem with the empirical standpoint that government spending induces a low-efficiency level among African countries [50, 57] though this does not support theories of government spending as a potential tool for high human development. For sensitive checks, the model without the inclusion of rent resources and colonial legacy was estimated. The result shows that there is a negative and significant relationship between government spending and efficiency level. This could be a reason for most African countries are within the range of low human development index. High spending profiles by most African governments do not potent high socio-economic development. This explains huge government spending among African countries has not been inclusive of human development as against theoretical standpoints.

Meanwhile, the size of African countries' economies has a positive and significant relationship with government spending efficiency level. By implication, as the size of an economy increases so is the rise of government spending efficiency. The same result has been found by Wagner theory and empirical standpoint Fonchamnyo and Sama [34]. This reveals that economic activities remain potential tool for inclusive development among African countries. Meanwhile, this result may not hold for the generality of Africa owing to individual specific attributes. In addition, robustness model found out out that there is a negative but insignificant relationship between the size of an economy and government spending efficiency level in Africa. The negative but insignificant influence could be due to some peculiar attributes attached to countries in the African economies. However, economic growth potent positive and significant influence on efficiency level irrespective of models estimated as such commands high human development among countries in Africa. This could be a reason for most emerging economies in the world are among the countries with high human development indicators.

On the institutional variables, findings show that control of corruption and efficiency level is positively but insignificantly related across models except on the correction for cross-sectional dependence which is significant at 1% level. This explains that high control of corruption commands high human development. Semantically, the low human development indicator among most African countries is due to the high corruption index among African countries. By implication, as African countries' control of corruption rises so is the government spending efficiency. Additionally, spending effectiveness and efficiency levels are negatively and significantly related across all models. By implication, government spending ineffectiveness creates a high inefficiency level. This is in tandem with [64, 76] that explained weak institutions as the inhibiting factor for human development growth in Africa.

Furthermore, resource rent and efficiency levels are negatively related. By implication as more rent resources is discovered so is the low level of government spending efficiency. This suggests that the effect of rent resources could pose danger to the African governments' spending efficiency when issues with spending-development nexus are collectively addressed. However, rent resources become positively related to government spending efficiency when African countries' peculiar attributes are considered and issues with cross-sectional dependence are corrected. This result could be due to diversity in a system of government and differences in unobserved attributes among African countries. This explains a unique way of addressing human development growth among countries in Africa.

Interestingly, colonial legacy and government spending efficiency are positively and significantly related across models. Impliedly, an increase in the years of colonial legacy brings about a rise in government spending efficiency. The more African countries become reform their institutions the higher the possibility of improvement in efficiency in public spending. The reason could be that there will be neutralization of the system of public sector operations and bureaucratic administrations in terms of public services, education, judiciary, financial institutions, and a host of other colonial masters handed down. In Table 9, the relationship between government spending efficiency and its drivers remains sacrosanct except with institutional variables and rent resources. Impliedly, there is no significant changes across African countries, resource-rich and non-resource-rich region.

In Table 9, there is a negative and significant relationship between control of corruption and efficiency level for resource-rich regions. By implication, as control of corruption rises, there is a fall in the efficiency of government spending among resource-rich regions. This suggests that control of corruption among resource-rich regions of Africa has an indirect effect on the efficiency level. The reason could be that most countries in resource-rich regions mobilize resource rent towards rent-seeking behaviour and as such leads to an increase in government spending profile without a corresponding rise in human development. However, among the resource-poor countries in Africa, control of corruption and government spending efficiency is positively and significantly related. The reason could be that high human development becomes the target within resource-poor regions and, as such, available resources are judiciously used rather than rent-seeking behaviour. Likewise, across both resource-rich and non-resource-rich regions, government effectiveness has a positive and significant influence on the efficiency level of government spending. The implication is that as government becomes effective in the quality of policy formulation and implementation, efficiency of spending becomes inevitable across both regions.

Finally, resource rent and efficiency in spending are negatively and significantly related to the resource-rich region while they are positively and significantly related to the non-resource-rich region. This finding revalidates the resource-cause hypothesis among selected African countries. Impliedly, naturally resource endowed countries have a slim chance of growing human development which could be evidenced in Nigeria, Niger, Guinea, Sierre-leone, Mozambique, and Uganda.

## Conclusion and policy implications

This paper has estimated the scope for government spending efficiency across selected African countries, resource-rich and non-resource-rich regions. It builds advances in the literature by applying SFA techniques to a high-quality cross-country government spending dataset and with an increased focus on the determinants of government spending efficiency. Our results suggest that resource rent, colonial legacy, institutional variables, size of an economy, and government spending all play a significant role in determining the extent to which a country's human development index reaches its potential and the efficiency of government spending. The results of the model show that the major deviation from the Cobb–Douglas function is attributed to inefficiency across the sample size. The much variation of the error term is found to be accounted for by the technical inefficiency rather than the noise effect. Thus, the greater percentage of the deviation in the error component is due to the technical inefficiency and provides an avenue to examine the factors that cause variation in government spending efficiency in Africa. However, the difference between the actual result and the observed result is attributed to the noise effect from the composite error term. Hence, the statistical significance of the estimate of *gamma* shows that the model is a good predictor of technical inefficiency and consistent with the extant studies [8, 12, 28, 74].

The study reveals that the exploration of natural resources could provide an improvement to government spending efficiency across African countries particularly the non-resource-rich region and at the same time improve the resources available for the government to spend. In addition, local autonomy will encourage the abundance of natural resources and increase resources available for government expenditure profile for each country. There is a need for the government in Africa to target inclusiveness, particularly to achieve socio-economic outcomes.

Furthermore, the existence of a relationship between drivers of government spending efficiency is concluded as dependent upon each African country's political independence, geographical location, economic independence, and the threat of terrorism and war that it faces which are measured through colonial legacy, rent resource, size of an economy, institutional variables, and government spending. Moreover, the study revealed how each driver influences the government spending efficiency. By implication, effective resources utilization and a strong institutional framework are potential drivers of spending efficiency in African economies. Meanwhile, it could also be seen that legacy received from colonial rulers have had a great influence on the nature of public sector operations. Hence, any distortion of the economy is a result of self-interest and personal gain in the management of public resources.

## Data Availability

The data are freely available on World Development Indicator and Worldwide Governance Indicator, published by World Bank.

## Abbreviations

NAC: North Africa countries
SAC: Southern African countries
CEN: Central African countries
WAC: West African countries
EAC: East African countries
GDP: Gross domestic product
HIV: Human immunodeficiency virus
AIDS: Acquired immunodeficiency syndrome
COVID: Corona Virus disease
SFA: Stochastic frontier analysis
FDH: Free disposal hull
DEA: Data envelopment analysis
TE: Technical efficiency
TFE: Total fixed effect
FEM: Fixed effect model
REM: Random effect model
PSCE: Panel corrected standard error
GLS: Generalized ordinary least square
NRC: Resource rent and colonial orientation.
IMF: International monetary fund
UNDP: United Nation Development Programme
CD: Cross-sectional dependence
